# Case Report: Clinical and molecular features of a radiosensitive autoimmune polyendocrine syndrome type 1 patient with oral carcinoma

**DOI:** 10.3389/fgene.2025.1588108

**Published:** 2025-10-21

**Authors:** Asma Chikhaoui, Houda Hammami-Ghorbel, Dorra Najjar, Semia Zarraa, Safia Yahiaoui, Davor Lessel, Houda Yacoub-Youssef

**Affiliations:** ^1^ Laboratory of Biomedical Genomics and Oncogenetics (LR16IPT05), Institut Pasteur de Tunis, Université Tunis ElManar, Tunis, Tunisia; ^2^ Department of Dermatology, Habib Thameur Hospital (LR12SP03), Faculty of Medicine, University of Tunis ElManar, Tunis, Tunisia; ^3^ Department of Radiotherapy, Salah, Azaiz Institute, Tunis, Tunisia; ^4^ Institute of Human Genetics, University Regensburg, Regensburg, Germany; ^5^ Institute of Clinical Human Genetics, University Hospital Regensburg, Regensburg, Germany

**Keywords:** autoimmune polyendocrine syndrome type-1, cerebral cavernous malformations, oral carcinoma, radiation-induced cytotoxicity, MAP3K3, AIRE

## Abstract

Autoimmune polyendocrine syndrome type-1 (APS1), also known as autoimmune polyendocrinopathy–candidiasis–ectodermal dystrophy (APECED), is an autoimmune genetic disease characterized by multiple endocrine disorders, chronic mucocutaneous candidiasis, and various ectodermal defects. Untreated candidiasis can increase the risk of oral cancer due to recurrent fungal infections. Radiotherapy is a curative option that can trigger an antitumoral response. However, exaggerated radiation-induced cytotoxicity can hinder this curative modality. APECED is caused by loss-of-function mutations in the autoimmune regulator *AIRE* gene, with only a few cases reported in Tunisia. In this study, we report the clinical, genetic, and molecular characteristics of a patient with APECED syndrome. This patient was initially referred for genetic inquiry due to extreme sensitivity to radiotherapy after developing oral squamous-cell carcinoma. Whole-exome sequencing (WES) was performed to identify disease-causing mutations. A set of candidate genes was further analyzed using real-time quantitative polymerase chain reaction (RT-qPCR) to explore the possible underlying interaction between the detected variant and altered gene expression in inflammatory pathways. We report a loss-of-function, germline, homozygous variant in the *AIRE* gene associated with APECED syndrome and a gain-of-function variant in mitogen-activated protein kinase kinase kinase 3 (MAP3K3), previously identified in patients with cerebral cavernous malformations (CCMs). Unexplained inflammatory and biochemical manifestations, including increased leukocyte, neutrophil, and C-reactive protein (CRP) levels, were noted. MAPK signaling is organized as a three-tier cascade, in which MAP3Ks activate MAP2Ks, which, in turn, activate MAPKs (ERK, p38, and JNK). These pathways regulate key cellular processes, such as proliferation, differentiation, and stress responses, with each kinase having distinct substrate specificity. Analysis of candidate gene expression interacting with the two key genes indicated the overexpression of *p38*, *TNF-α*, and *STAT3*, which may be associated with these manifestations. Our results underline the impact of WES in clinical diagnosis and confirm the impact of the identified variants on disease manifestation. We also suggest that the co-occurrence of APECED syndrome and a possible variant causing CCMs may be involved in the poor survival of atypical oral carcinoma cases and radiation-induced cytotoxicity.

## 1 Introduction

Autoimmune polyendocrine syndrome type-1 (APS1), also known as autoimmune polyendocrinopathy–candidiasis–ectodermal dystrophy (APECED, OMIM #240300), is a monogenic disease characterized by multiple autoimmune disorders and chronic (or recurrent) candidiasis of the skin, nails, and mouth, which increases the risk of cancer (Rautemaa et al., 2007). APECED is caused by biallelic loss-of-function, pathogenic mutations in the autoimmune regulator *AIRE* gene, which encodes the homonymous (AIRE) protein, a transcription factor responsible for the negative selection of maturing, self-reactive thymocyte clones. It is a rare disease, with a prevalence of 1:100,000 (Anderson and Su, 2011).

Only three Tunisian patients with APECED have been previously reported, and they showed unusual clinical onset but no association with oral cancer (Ach et al., 2023; Arousse et al., 2018; Younes, 1970).

Radiation therapy is one of the most important curative procedures for managing oral squamous-cell carcinoma. Its success depends on various factors, including immunological status and several biological parameters. Alterations in the intrinsic microenvironment can lead to a high rate of severe radiotherapy-related toxicity, local recurrence, or metastatic recurrence (Li et al., 2023).

In this study, we aimed to investigate the clinical picture and genetic background of a Tunisian patient who exhibited various endocrine manifestations and a history of chronic candidiasis that was observed only after the development of oral squamous-cell carcinoma. The patient also exhibited low tolerance to radiotherapy and a generalized inflammatory state. Whole-exome sequencing revealed that the patient harbored a homozygous mutation in the autoimmune regulator (*AIRE)* gene. At the same time, a heterozygous mutation of the mitogen-activated protein kinase kinase kinase 3 (*MAP3K3*) gene, which encodes the homonymous protein MAP3K3, was found. This genetic profile was accompanied by a gene activation pattern that could contribute to the above-mentioned immune responses.

## 2 Materials and methods

### 2.1 Patient

Patient data were collected during the last clinical examination and diagnostic reasoning in 2022. Written informed consent was obtained for molecular testing and medical photography. The study was conducted in accordance with the World Medical Association Helsinki Declaration and was approved by the Institut Pasteur de Tunis (IPT) Biomedical Ethics Committee (ethical approval reference 2017/31/I/LR16IPT05/V2).

### 2.2 DNA extraction

Genomic DNA was isolated from peripheral whole blood using a FlexiGene DNA Kit (QIAGEN; Cat. No./ID: 51206, Hilden, Germany), according to the manufacturer’s instructions. DNA quality was assessed using a DS-11 NanoDrop Spectrophotometer (DeNovix Wilmington, USA).

### 2.3 Whole exome sequencing

For next-generation sequencing (NGS), whole-exome sequencing (WES) was performed on two lanes of a NextSeq 2000 Sequencing System (Illumina San Diego, CA, USA) after enrichment of exonic and splice-site sequences using the Twist Human Core Exome Kit (Twist Bioscience San Francisco, CA, USA). More than 125 million reads were mapped to the hg19 human reference genome sequence. Approximately 98.5% of the exome had 20-fold coverage. Data analysis of the filter-passed reads was performed using VarSome Clinical, a CE VD-certified and HIPAA-compliant platform. The criteria for considering a variation were >6 reads, a Phred-scaled quality score >15, a population minor allele frequency (MAF) < 1%, <10-time occurrence in our in-house database, and >15% of the reads supporting the allele.

Variant filtering was performed based on autosomal dominant, autosomal recessive, and X-linked inheritance models.

### 2.4 Sanger sequencing

To verify the prospective pathogenic mutations detected through WES, Sanger sequencing was performed using an ABI 3100 DNA Sequencer (Applied Biosystems Weiterstadt, Germany).

### 2.5 Real-time quantitative polymerase chain reaction analysis of candidate gene expression

Total RNA extraction from peripheral blood mononuclear cells (PBMCs) was performed using Invitrogen TRIzol Reagent 100 (Thermo Fisher Scientific USA) for the patient and an age- and sex-matched healthy subject. cDNA synthesis was performed using 1 μg of RNA via Invitrogen SuperScript II Reverse Transcriptase (Thermo Fisher Scientific). Real-time quantitative polymerase chain reaction (RT-qPCR) was performed using a set of in-house primers with amplification using SYBR Green Master Mix (Roche Life Science Penzberg, Germany). Candidate genes were selected from available primers for which *AIRE* and *MAP3K3* displayed maximum interaction using the online tool genenetwork.nl. Gene expression analysis was performed using the comparative CT (ΔΔCT) method with LightCycler 480 Software (Roche Life Science) in triplicate, with normalization to the housekeeping gene *RPLP0*.

## 3 Case description

### 3.1 Clinical description

This study describes a patient born through normal delivery within an endogamous marriage and referred to the hospital for the detection of oral squamous-cell carcinoma. Genetic and clinical investigations were performed during the first manifestation of radiosensitivity after a radiotherapy session.

**FIGURE 1:**
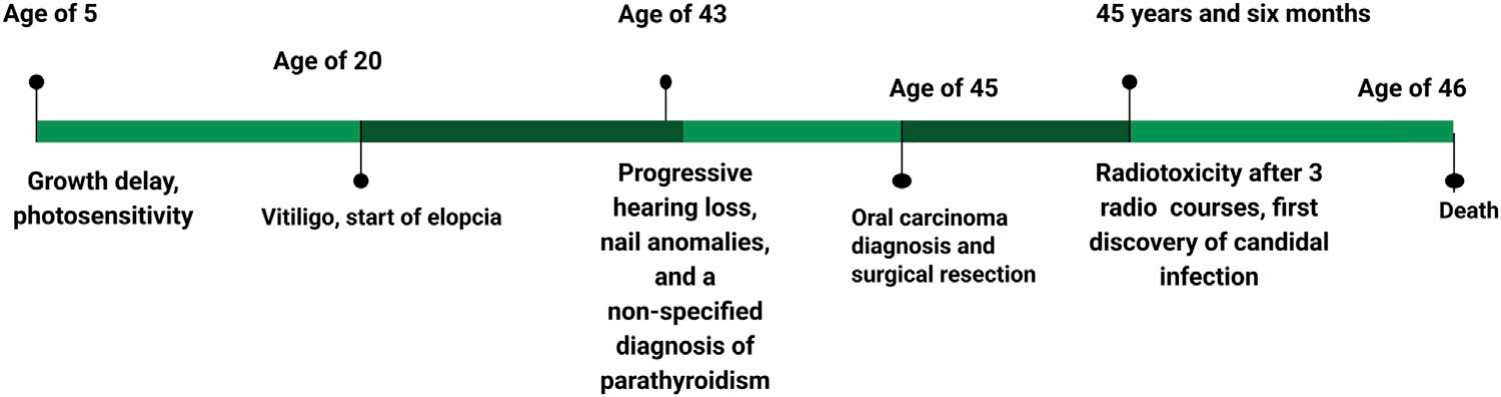
Chronology of notable events throughout the patient’s history.

Anamnesis revealed that the patient had been diagnosed with growth delay since childhood. Patches of vitiligo appeared at the age of 20 years, and progressive hearing loss was noted in the 2 years preceding the observation (Figure 1). Strangely, oral *Candida* infection was observed in the 5 months preceding the genetic inquiry.

The investigation took place after three sessions of radiotherapy. The patient was 46 years old and appeared as an extremely emaciated subject, with a significantly low height (150 cm, <1st pc, −4.43 SD) and weight (32 kg, <1st pc, −7.51 SD). His face and body showed folds of cutis laxa caused by the loss of subcutaneous adipose tissue. The patient had alopecia universalis, and the nails had a sandpaper-like texture. He complained of photophobia, and an ophthalmoscopic examination revealed interstitial keratitis.

Endocrinological assessment revealed idiopathic hypoparathyroidism, primary adrenal insufficiency, and atrophic gastritis with related pernicious anemia. Coexisting azoospermia without any genital anomaly was also noted. Laboratory analyses highlighted a generalized inflammatory state, characterized by leukocytosis with neutrophilia, thrombocytosis, and increased serum C-reactive protein (CRP), which made him unfit for chemotherapy. Hyperphosphatemia was observed, but serum calcium levels were higher than the normal range.

The patient underwent three courses of radiotherapy (volumetric modulated arc therapy), to which he showed low tolerance, as evidenced by restricted mouth opening (trismus), stomatitis, and dry mouth.

Radiotherapy was administered at a reduced dose of 60 Gy, with 1.8 Gy per day during hospitalization, to benefit from close clinical monitoring. The patient developed early grade II radiodermatitis according to the CTCAE international grading scale at a dose of 10 Gy, which progressed owing to local treatment. Consequently, the patient showed spectacular clinical tumor regression at a dose of 44 Gy, but this was associated with radiomucitis and grade III radiodermatitis complicated by bacterial and candidal superinfection, requiring temporary discontinuation of radiotherapy. Local treatment with oral antibiotics and anti-inflammatory therapy was initiated. As the adverse effects related to toxicity had improved, radiotherapy was resumed after a one-week break, but it was definitively discontinued at a dose of 50 Gy due to the recurrence of Grade III toxicity.

The patient was in complete clinical remission at the time. An objective radiological evaluation was scheduled 2 months after the end of radiotherapy. The patient was observed 7 days, 15 days, and 1 month after the end of radiotherapy. Clinically, there was no evidence of tumor recurrence, and toxicity, particularly skin and mucosal toxicity, improved significantly. The patient was scheduled for a clinical and radiological check-up 3 months after the end of radiotherapy, at which point we were informed of his death. He was taken to the emergency room due to an altered state of consciousness.

**FIGURE 2:**
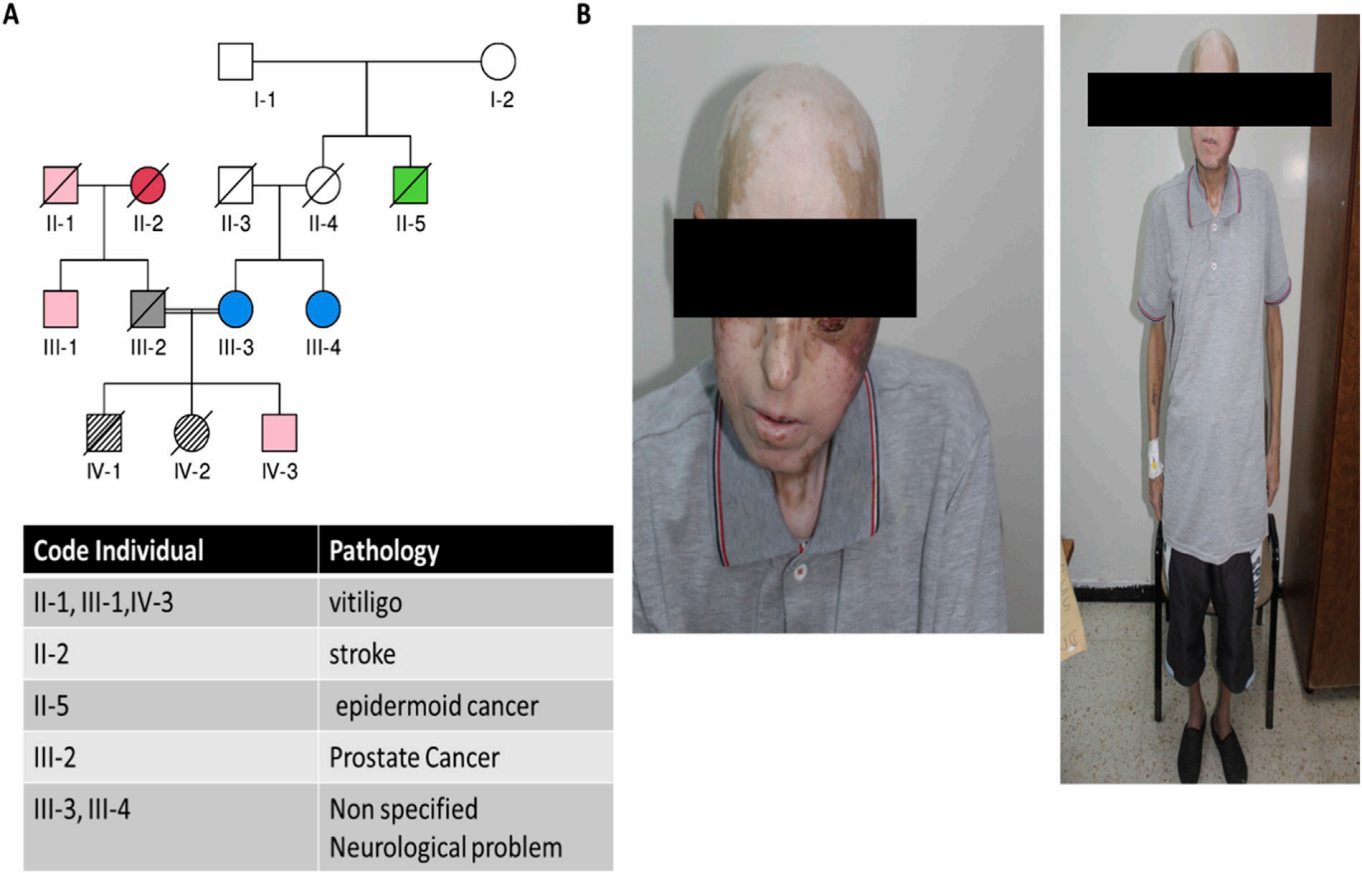
Clinical and genealogical data from the studied patient. **(A)** Pedigree showing the proband and different pathologies within the family; **(B)** clinical images of the patient showing alopecia, vitiligo, and the development of oral carcinoma.

The family history revealed that the patient’s sister had a similar clinical picture during her lifetime, and her death was likely attributed to a stroke. Several family members had late-onset vitiligo, and some cases of unspecified neurological issues and prostate cancer were also reported (Figures 2A,B).

### 3.2 Genetic findings

Given the features of the patient’s phenotype and family history, we hypothesized that a genetic disease underlies his condition. Therefore, WES was performed, and the results were validated using Sanger sequencing.

WES showed that the patient harbored a homozygous mutation in *AIRE*, c.931delT (NM000383.4), leading to an *AIRE* variant (p.Cys311ValfsTer67). Another heterozygous possible germline mutation (c.1323C>G, NM_002401.5, MAF <0.00001%) was found in *MAP3K3* at a deep coverage rate (being identified in 269 out of 489 reads), which caused the *MAP3K3* variant (p.Ile441Met). Both protein variants were hypothesized to be pathogenic according to various prediction tools and previous publications. Sanger sequencing initially confirmed only the *AIRE* mutation. *MAP3K3* was consequently detected after the use of several primers due to a probable allelic dropout phenomenon (data not shown).

### 3.3 Assessment of candidate gene expression and potential interactions with mutated genes

We first evaluated *AIRE* and *MAP3K3* expressions to assess the functional impact of the two detected mutations. RT-qPCR analysis showed a significant decrease in *AIRE* mRNA (fold change 0.06) compared to the healthy control. In contrast, the *MAP3K3* mutation seemed to increase the related mRNA amount (fold change 4.282).

**FIGURE 3:**
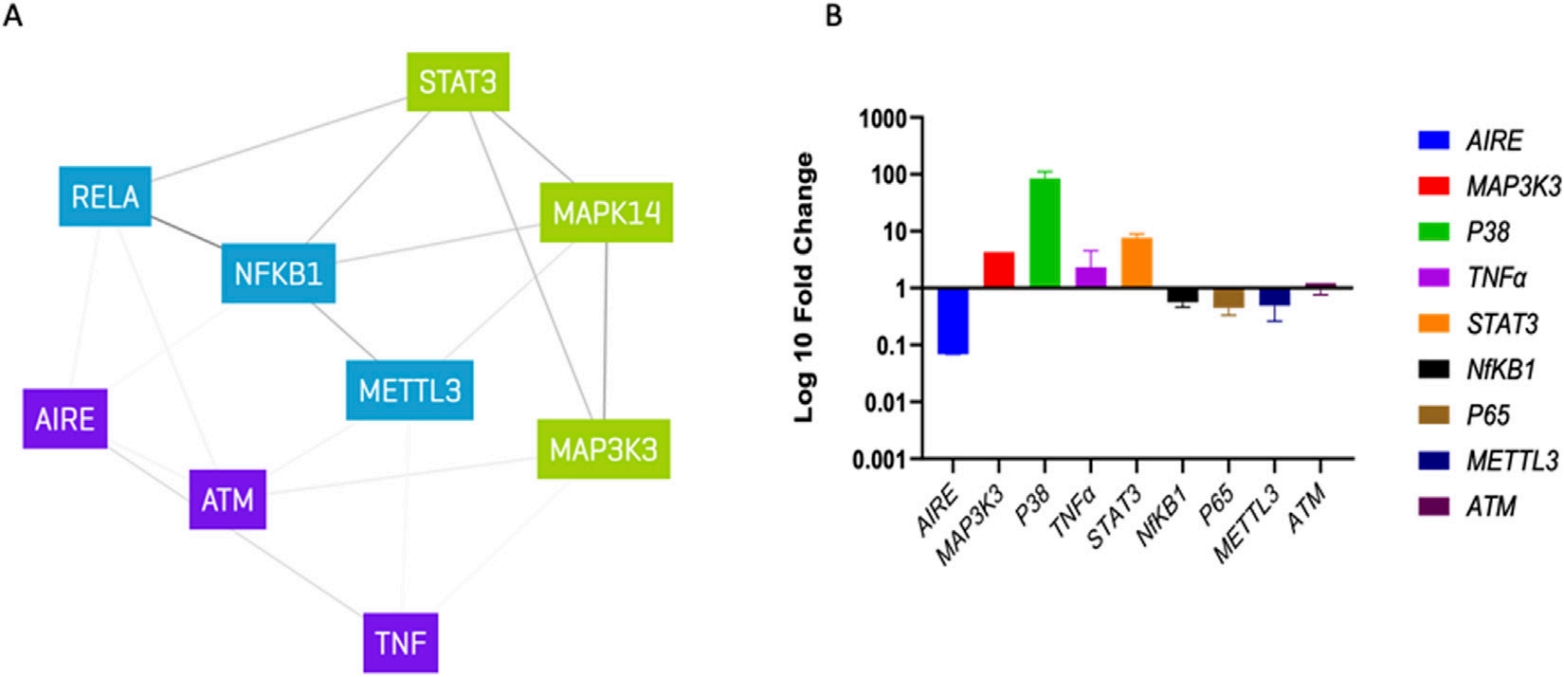
Gene expression analysis: **(A)** list of candidate gene interaction with MAP3K3 and AIRE determined via genenetwork.nl, different colors indicating possible clusters. **(B)** Log 10-fold change qPCR analysis normalized to healthy control.

Since *AIRE* deficiency and *MAP3K3* overexpression did not explain the patient’s low tolerance to radiotherapy treatment, we evaluated the expression of a panel of candidate genes, namely, P38Alpha/mitogen-activated protein kinase 14 *(P38α/MAPK14)*, tumor necrosis factor *(TNF)*, signal transducer and activator of transcription *3 (STAT3)*, nuclear factor kappa B subunit 1 *(NF-κB1)*, *P65/RELA proto-oncogene*, *NF-κB subunit (P65/RELA)*, methyltransferase 3, N6–adenosine–methyltransferase complex catalytic subunit *(METTL3*), and ATM serine/threonine kinase *(ATM)* (Figure 3A), whose corresponding proteins could be related to inflammatory response and DNA repair pathways and which could ultimately play a role in low tolerance to radiotherapy. As a result, three genes, *P38α/MAPK14*, *TNF*, and *STAT3*, were overexpressed in the patient (fold change >2), while an underexpression of *P65/RELA* and *METTL3* (fold change <0.5) was noted. The expression levels of NF*-κB1* and *ATM* were similar to those of healthy subjects (Figure 3B).

## 4 Discussion

Autoimmune polyendocrine syndromes (APSs) are a heterogeneous group of diseases characterized by the co-occurrence of at least two autoimmune-mediated endocrinopathies and are classified according to the affected organs. APS1, commonly known as APECED, is an autosomal recessive monogenic disease caused by pathogenic mutations in the *AIRE* gene, which is in the chromosomal region 21q22.3. The related protein, AIRE, is a gene transcription factor involved in the negative selection of maturing, self-reactive thymocyte clones (Bruserud et al., 2016). Other AIRE-expressing cell lineages have been described, but the extent and function of AIRE in extrathymic tissues have not been fully elucidated (van Laar et al., 2022). AIRE contains various functional domains, such as a caspase activation and recruitment domain (CARD), a nuclear localization signal (NLS), a SAND domain, and two plant-homeodomain fingers, PHD1 and PHD2, the latter being responsible for the activation of gene transcription (Perniola and Musco, 2014; Yang et al., 2013).

Tunisian APECED patients described in previous reports exhibited the pathogenic protein variants Ala58Asp and Arg257Ter, lying in the CARD and SAND domains, respectively (Ach et al., 2023; Arousse et al., 2018). Despite an atypical onset, both patients showed endocrinological deficiencies, dermatological manifestations, and ectodermal dystrophies typical of the APECED picture, but no oral cancer was observed. Our patient’s AIRE variant was located in PHD1 and has previously been reported in European APECED patients (Garelli et al., 2021; Mezgueldi et al., 2015; Bjørklund et al., 2022). Using RT-qPCR, we confirmed the effects of the *AIRE* mutation, leading to poor gene expression in PBMCs, as the encoded truncated protein was likely to promote nonsense-mediated mRNA decay.

Some studies have highlighted that certain AIRE mutations exert pathogenic effects in a dominant fashion and show variable penetrance and degrees of autoimmunity (Oftedal et al., 2023). The occurrence of late-onset vitiligo in some members of the patient’s family resembled this condition, but the unavailability of genetic data prevented us from pursuing this hypothesis.

WES identified a second gene mutation involving the *MAP3K3* gene, located in the chromosomal region 17q23.3. The related protein is a serine/threonine-specific protein kinase belonging to the family of mitogen-activated protein kinase kinase kinases (MAP3Ks), which activate mitogen-activated protein kinase kinases (MAP2Ks), which, in turn, activate mitogen-activated protein kinases (MAPKs), forming a three-tiered kinase cascade in MAPK signaling (Chen et al., 2001). This variant was not first validated via Sanger sequencing due to allelic dropout, which represents a limitation of regular Sanger sequencing, as suggested by previous studies on other disorders (Shestak et al., 2025).

MAPK pathways regulate various cellular processes, such as proliferation, differentiation, and stress responses. Furthermore, each component targets specific downstream kinases, with MAP3Ks such as Raf and ASK1, MAP2Ks including MEKs and MKKs, and MAPKs such as ERK, p38, and JNK, exhibiting diverse substrate specificity and function (Peterson et al., 2022). Under ordinary conditions, MAP3K3 acts upstream of the P38α/MAPK14 pathway and is involved in cellular response to external stimuli (Suddason and Gallagher, 2015). It has been demonstrated that overexpression of MAP3K3 plays a role in regulating NF-κB signaling through TNF in ovarian carcinoma (Zhang et al., 2019). Above all, various reports highlighted an association between the detected somatic mutation in MAP3K3 and the occurrence of CCMs, which, in rare cases, can cause hemorrhagic stroke (Ren et al., 2023). In our case, given the complication of squamous-cell carcinoma, the clinicians did not suspect the presence of vascular anomalies. The presence of a familial history of stroke and neurological problems suggests a possible interaction with the *AIRE* mutation, which may act as a genetic driver for the development of somatic *MAP3K3* alterations, and raises questions about whether the variant is somatic or inherited. In addition, the lack of detailed information on the event and the unavailability of neuroimaging precluded any type of verification. Furthermore, unfortunately, we were unable to reach any relative of the studied patient. As a course of action, we suggest that collaborating clinicians investigate genetic variants in similar cases originating from the same region.

An undesirable aspect of the patient’s clinical course was the development of oral squamous-cell carcinoma, which probably masked the serum calcium dysregulation of hypoparathyroidism (Iwase et al., 2001). Oral squamous-cell carcinoma is, like most cancers, a multifactorial disease caused by the interaction of genetic, epigenetic, and environmental factors. In Tunisia, the disease displays an increasing incidence (18.3/100,000/year) and morbidity (Mnejja, 2025). In patients with APECED, the enhanced risk of oral (and esophageal) squamous-cell carcinoma is related to chronic/recurrent mucocutaneous candidiasis. As innate immunity is preserved (Perniola et al., 2008), autoimmune mechanisms, such as the production of anti-interferon and anti-cytokine autoantibodies, have been implicated in the dysregulation of adaptive immunity (Meloni et al., 2008). Moreover, it has been demonstrated that *AIRE* is induced in cancer cells and supports cancer-related gene expression (Nguyen et al., 2020). Therefore, it would have been more suitable to investigate gene expression in a tumor biopsy from the studied individual. Unfortunately, the patient died before we could ascertain this. Considering the clinical manifestations observed in this patient, it is advisable to assess the level of *AIRE* gene expression in patients who are developing cancer, due to its probable association with tumorigenesis and radiosensitivity.

Treatment options for oral squamous-cell carcinoma vary according to the disease stage and include surgery, chemotherapy, radiotherapy, or a combination of the three. The evaluation of the clinical and biological parameters of this patient made him unfit for chemotherapy, in accordance with previous criteria suggested by clinical consensus, such as his hematological status (Falco et al., 2021). Radiotherapy may be associated with side effects (in approximately 39% of treated patients) and death risk (up to 3%–4% of patients) (De Ruysscher et al., 2019). Our patient experienced early-onset restricted mouth opening (trismus), stomatitis, and dry mouth (usual signs of late-onset intolerance), which led to the decision to discontinue the treatment. The patient underwent genetic and molecular investigations due to the hindrance of proper alimentation following the cessation of treatment, coupled with an increased awareness stemming from a complex family history of cancer and neurological disorders.

The application of next-generation sequencing facilitated diagnosis in our case. Initially, laboratory findings related to autoimmune polyendocrine syndrome were overlooked due to the pronounced accelerated aging phenotype observed in the patient (cutis laxa, hearing loss, and subcutaneous fat loss) and the absence of *Candida* infection during the first period of diagnosis. This led us to initially hypothesize a DNA repair disorder, which is generally characterized by sensitivity to radiotherapy (Pollard and Gatti, 2009). Therefore, the exact causes of unexplained accelerated aging in the patient and his deceased sibling remain unknown.

To explain the patient’s low tolerance to radiotherapy, we assessed a panel of candidate genes encoding proteins involved in inflammatory and DNA repair processes. Interestingly, we observed high expressions of *STAT3*, *P38α/MAPK14*, *and TNF*. The *STAT3* transcription factor acts as a regulator of mitochondrial function during early carcinogenesis by interacting with the Ca^2+^ channel. *STAT3* has proliferative and anti-apoptotic effects that play a role in inflammation and resistance to anti-cancer therapies, even in oral squamous-cell carcinoma. The other two overexpressed genes encode proteins with well-recognized roles in the induction and action of inflammatory cytokines (Zhang et al., 2019).

In contrast, the expression levels of *P65/RELA* and *METTL3* were significantly reduced. The formation of N6–methyladenosine is the most abundant mRNA modification and is catalyzed by a methyltransferase complex, in which METTL3 is the only catalytic subunit. This important process controls gene expression and DNA damage signaling. It has been shown that, upon irradiation, the expression of METTL3 (the corresponding murine gene) is suppressed in xenograft tumors (Sun et al., 2023), similar to what was observed in our patient.

Finally, ATM has been identified as a candidate gene that interacts with AIRE. The encoded protein, ATM, is a DNA-dependent protein kinase that may affect the risk of radiotherapy-induced side effects (Abramson et al., 2010). In any case, we found that *ATM* expression was unaffected; therefore, it was not involved in the clinical course.

Even when constrained by a limited number of cases, expanded molecular profiling often serves as a driver for exploratory research concepts, particularly in the context of atypical phenotypes associated with rare disorders. This is notably achieved through gene expression analysis, as stated by various studies, since it offers a deeper understanding of the impact of pathogen variants predicted solely through algorithmic prediction (Najjar et al., 2022; Nezu et al., 2023).

In conclusion, we described a Tunisian patient with APECED, whose clinical course was complicated by the occurrence of oral squamous-cell carcinoma. WES revealed a homozygous mutation in *AIRE* and a heterozygous variant in *MAP3K3*, whose significance and impact remain unclear due to the absence of more detailed neuroradiological investigations. To explain the poor tolerance to radiotherapy and the development of unwelcome side effects, the expression of a panel of genes related to inflammatory processes and tissue repair was investigated, providing valuable information. Our study highlights the importance of a comprehensive approach to the genetic investigation of inflammatory diseases, particularly autoimmune diseases, to identify more appropriate treatment options for patients with rare and complex syndromes. Our report, acknowledging that it pertains to an individual case, outlines several limitations concerning the determination of the precise cause of the patients’ death. Furthermore, it highlights the temporal delay in uncovering cerebral vascular anomalies, which, if identified at a more opportune moment, might have facilitated improved management of the patient’s condition.

## Data Availability

The raw data supporting the conclusions of this article will be made available by the authors, without undue reservation.
